# Identification of type I interferonopathies using blood interferon signature: the experience of a pediatric rheumatology center

**DOI:** 10.1186/1546-0096-13-S1-P142

**Published:** 2015-09-28

**Authors:** S Volpi, E Santori, P Picco, G Rice, R Caorsi, A Martini, Y Crow, F Candotti, M Gattorno

**Affiliations:** 1 Istituto G. Gaslini, U.O. Pedatria 2, Genova, Italy; 2 Lausanne University Hospital, Allergy and Immunology, Lausanne, Switzerland; 3 University of Manchester, Manchester Academic Health Science Centre, Genetic Medicine, Manchester, UK; 4 Institute Imagine, University Paris Decartes, Paris, France

## Question

To test blood interferon signature as a screening tool for type I interferonopathies in children with early-onset SLE and vasculopathy.

## Methods

We collected blood samples from a cohort of pediatric rheumatologic patients and scored them according to a qPCR based IFN gene signature assay. Expression of 6 type I IFN-related genes (*IFI27, IFI44L, IFIT1, ISG15, RSAD2, SIGLEC1*) was quantified by standard RTPCR techniques. An IFN score was calculated for each patient using the median fold change of gene expression related to a healthy control. Patients were selected based on the presence of the following features: i) atypical or incomplete SLE-like symptoms occurring in infancy or in preprepubertal age; ii) vasculopathy (skin ulcers, chilblains, strokes) iii) panniculitis with or without lypodystrophy iv) interstitial lung disease in the context of systemic inflammation.

## Results

We screened 24 patients from the rheumatology unit of the Gaslini Children's Hospital, Genova (Italy). 14 out of 24 patients had a positive signature with an IFN score ranging from 3.5 to 23,7. Based on the clinical presentation and the result of the IFN score we further analyzed two patients for mutations affecting *TMEM173* gene: in one patient we identified the previously reported V155M variant and in the second patient we identified a new variant whose pathogenic significance is currently under study. Molecular screening for genes associated to type I Interferonopathies and whole exome sequencing is ongoing for selected patients.

## Conclusions

Several studies have shown how mutation in genes involved in type I interferon (IFN) pathway can be responsible of severe inflammatory syndromes such as Aicardi-Goutières syndrome (AGS) and spondyloenchondrodysplasia with immune disregulation (SPENCD). STING associated vasculopathy with onset in infancy (SAVI) and a familial inflammatory syndrome with systemic lupus erythematosus (SLE)-like manifestations are among the last clinical phenotypes that have been linked to an increase of type I IFN - in both cases as a result of *TMEM173* gain of function dominant mutations. With the present ongoing study we anticipate that testing blood for an interferon signature is a valuable screening tool for the identification of type I interferonopathies in selected children with rheumatic diseases.

